# Integration and dosimetric validation of a dynamic collimation system for pencil beam scanning proton therapy

**DOI:** 10.1088/2057-1976/ad02ff

**Published:** 2023-10-25

**Authors:** Nicholas P Nelson, Wesley S Culberson, Daniel E Hyer, Theodore J Geoghegan, Kaustubh A Patwardhan, Blake R Smith, Ryan T Flynn, Alonso N Gutiérrez, Thibault Boland, Patrick M Hill

**Affiliations:** 1 Department of Medical Physics, School of Medicine and Public Health, University of Wisconsin—Madison, 1111 Highland Avenue, Madison, WI, 53705, United States of America; 2 Department of Radiation Oncology, University of Iowa Hospitals and Clinics, 200 Hawkins Drive, Iowa City, IA, 52242, United States of America; 3 Department of Radiation Oncology, Miami Cancer Institute, Baptist Health South Florida, 8900 N. Kendall Drive, Miami, FL, 331765, United States of America; 4 Ion Beam Applications (IBA), Louvain-la-Neuve, Belgium; 5 Department of Human Oncology, School of Medicine and Public Health, University of Wisconsin—Madison, 600 Highland Avenue, Madison, WI, 53792, United States of America

**Keywords:** proton therapy, collimation, Monte Carlo, dosimetry

## Abstract

**Objective.:**

To integrate a Dynamic Collimation System (DCS) into a pencil beam scanning (PBS) proton therapy system and validate its dosimetric impact.

**Approach.:**

Uncollimated and collimated treatment fields were developed for clinically relevant targets using an in-house treatment plan optimizer and an experimentally validated Monte Carlo model of the DCS and IBA dedicated nozzle (DN) system. The dose reduction induced by the DCS was quantified by calculating the mean dose in 10- and 30-mm two-dimensional rinds surrounding the target. A select number of plans were then used to experimentally validate the mechanical integration of the DCS and beam scanning controller system through measurements with the MatriXX-PT ionization chamber array and EBT3 film. Absolute doses were verified at the central axis at various depths using the IBA MatriXX-PT and PPC05 ionization chamber.

**Main results.:**

Simulations demonstrated a maximum mean dose reduction of 12% for the 10 mm rind region and 45% for the 30 mm rind region when utilizing the DCS. Excellent agreement was observed between Monte Carlo simulations, EBT3 film, and MatriXX-PT measurements, with gamma pass rates exceeding 94.9% for all tested plans at the 3%/2 mm criterion. Absolute central axis doses showed an average verification difference of 1.4% between Monte Carlo and MatriXX-PT/PPC05 measurements.

**Significance.:**

We have successfully dosimetrically validated the delivery of dynamically collimated proton therapy for clinically relevant delivery patterns and dose distributions with the DCS. Monte Carlo simulations were employed to assess dose reductions and treatment planning considerations associated with the DCS.

## 1. Introduction

Pencil beam scanning (PBS) proton therapy is a form of radiotherapy that consists of magnetically scanning millimeter-wide pencil beams to deliver therapeutic radiation treatments to tumors in successive energy layers [1, 2]. In PBS, however, treatment quality in terms of lateral target conformity is often limited by the system’s effective spot size at the patient surface [3-5]. To combat this issue, static apertures can be milled to conform to the projection of the largest energy layer in a given treatment to collimate the scanned field. While this does provide advantageous dosimetric benefits through the reduction of the effective spot size near the target edges, static collimators result in under-collimation of all other energy layers, an effect that is exacerbated for irregular shaped target volumes [6]. To address this, external dynamic collimation devices have gained interest in improving lateral conformity in PBS proton therapy [6-20]. These devices, categorized as per-field apertures, multileaf collimators, and sliding bar collimators, enable dynamically collimated proton therapy (DC-PT) [15].

One specific external collimation device, the Dynamic Collimation System (DCS), is being developed in conjunction with the IBA ProteusPlus system [13, 14, 20]. The Dynamic Collimation System (DCS) is a sliding-bar external collimation device that consists of four nickel trimmer blades that rapidly and independently move to intercept the scanning beam as it approaches the target edges. The DCS is equipped with an optional polyethylene range shifter to treat at depths below 4 cm. The current clinical DCS prototype utilizes trimmers that are 3 cm thick to fully attenuate 160 MeV protons and nickel to minimize neutron production [21]. The maximum energy was selected following the findings of Bues *et al*, who reported a loss of benefit with collimation at a depth of 17.5 cm [22]. The DCS is mounted on a telescoping snout that allows for varying trimmer-to-surface distances (TSD), defined as the distance between the bottom edge of the lowest collimating trimmer and the surface of the phantom.

Recent developments for this technology have consisted of the construction of a clinical prototype of the DCS [13] and a central axis alignment trimmer quality assurance device [23] as well as the development of a Monte Carlo model of the DCS mounted to the DN system [14]. More recently, an analytical algorithm has been developed to enable clinical treatment planning with the DCS [24-26]. While significant progress has been made in the development of the DCS, much of it has been confined to computational treatment planning studies and simplified experimental setups that do not fully represent clinical treatment scenarios.

The objective of this work is to provide an experimental basis of a complete collimated delivery from a clinically integrated DCS. To achieve this, the DCS control system will be integrated with the IBA beam scanning controller system, enabling the automated delivery of DC-PT with the DCS. Previous experimental studies have been limited to single beamlet irradiations or static trimmer configurations [8, 9, 12, 14, 27]. This work extends beyond those limitations by developing and simulating clinically relevant delivery patterns, parameterizing the expected dosimetric benefits of the DCS, and comparing the measured results with Monte Carlo simulations. In previous investigations, the impact of TSD on achievable penumbra has been evaluated for simple cubic fields [27]. However, this effect has yet to be addressed and experimentally verified for more clinically relevant dose distributions resulting from more complex delivery patterns. Additionally, this study explores the number of trimmer configurations requested per layer and considers the tradeoffs between dosimetric gains and delivery accuracy. By undertaking these simulation and experimental validation efforts, this research aims to bring DC-PT with the DCS closer to practical implementation, contributing to its potential clinical application.

## 2. Methods and materials

### 2.1. Treatment planning

A Monte Carlo-generated beamlet library and analytical plan creation techniques were employed to develop a set of uncollimated and collimated monoenergetic treatment fields consisting of one single energy layer. These fields were carefully designed to mimic clinically relevant delivery patterns that necessitate multiple trimmer positions per energy layer to achieve optimal treatment. The primary objective behind developing these fields was to facilitate the integration of the DCS control system with the IBA beam scanning controller. Additionally, these realistic target geometries served the purpose of offering valuable insights into the expected dose reductions in the normal tissue surrounding the target when utilizing the DCS system. The intention was to provide an experimental benchmark to healthy tissue sparing with the DCS.

**Figure 1.**
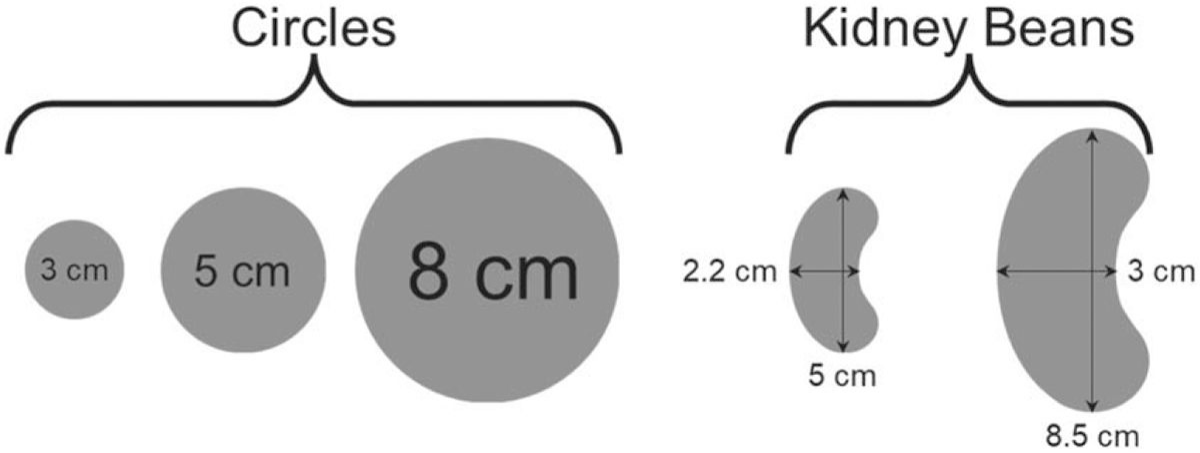
Illustration of target geometries evaluated *in silico*.

In this study, we considered circular shapes with varying diameters of 3 cm, 5 cm, and 8 cm, as well as two kidney bean shapes of different sizes for dosimetric characterization *in silico*, however, experimental efforts in this work focused only on the large kidney bean shape. Figure 1 provides an illustration of these shapes. The selection of kidney bean shapes was inspired from the AAPM Task Group 53 (TG-53) [28], which provides guidelines for quality assurance procedures related to treatment planning systems, including MLC testing. Circular targets with varying diameters were chosen due to their simplicity, generalizability, and their resemblance to an energy layer used for treating a spherical target.

#### 2.1.1. Monte Carlo beamlet library

The Dynamic Collimation Monte Carlo (DCMC) package [29] was employed to simulate uncollimated and collimated beamlets, which were used to populate pencil beam libraries for the beamlet selection and weight optimization algorithm. The DCMC package is an open-source extension library based on TOPAS [30] that incorporates a beam model of the IBA Dedicated Nozzle (DN) system at the Miami Cancer Institute, along with a model of the trimmer components used to simulate the DCS system. Previous work has already validated this model against DCS-collimated beamlets, both in the presence and absence of an external polyethylene range shifter [14].

Non-range shifted (NRS) and range shifted (RS) beamlets were generated to treat targets at depths ranging from 2 cm to 22.5 cm. For simulations at a depth of 22.5 cm, the trimmer thickness was increased within the Monte Carlo model from 3 cm to 4 cm to ensure full attenuation of protons with energies greater than 160 MeV. Table 1 provides a summary of the treatment depths and beams considered for the monoenergetic treatment fields developed. The range shifter is required for treatment depths below 4 cm, which corresponds to the range of the lowest available beam energy at the Miami Cancer Institute (MCI) of 70 MeV. For depths beyond 4 cm, both RS and NRS plans were evaluated because RS beamlets used for treating at depths of 5 cm and 10 cm exhibit a smaller spot size at isocenter compared to their lower-energy NRS counterparts (see table 1, $\sigma_{air}$ for 5 cm depth). Thus, this study also serves as an evaluation of the utility of the range shifter for depths beyond 4 cm, providing additional guidance to end users regarding its benefits. Additionally, the effect of the TSD on the achievable dosimetric benefits was evaluated at a depth of 5 cm using RS- and NRS-based deliveries, considering TSD values of 5, 10, and 15 cm.

The energy-specific beamlet libraries included an uncollimated beamlet and collimated beamlets with trimmer offsets of 1 and 2 mm for each trimmer, as shown in table 2. A minimum offset of 1 mm was selected based on the findings of Smith *et al* (2020) [31], who investigated the robustness of DC-PT with the DCS in terms of spot positioning, trimmer position, and mounting alignment uncertainties associated with the IBA universal nozzle system and concluded that implementing a minimum trimmer offset could improve the robustness in delivery with minimal impact on the high-dose conformity afforded by the DCS. The following assumptions were made within the beamlet library:

1. Beamlets can only experience collimation by a maximum of two trimmers.
2. Beamlets collimated by two trimmers simultaneously can only experience collimation by orthogonal trimmer pairs (e.g., any X trimmer with any Y trimmer, but not both X trimmers).
3. Symmetry is assumed between X and Y trimmers, allowing for beamlet kernels to be rotated and flipped to model collimation from an opposing trimmer (e.g., an X1-collimated beamlet can be flipped to model an X2-collimated beamlet).

TOPAS (Version 3.8.p1) Monte Carlo simulations were performed for the nine unique configurations (table 2) for each energy investigated. The dose to water was scored within a three-dimensional (3D) 1 mm isotropic dose grid embedded in a 40 × 40 × 40 cm^3^ water phantom, using 10^7^ histories per configuration. The default TOPAS physics modules (*g4em-standard_opt4*, *g4h-phy_QGSP_BIC_HP*, *g4decay*, *g4ion-binarycascade*, *g4h-elastic_HP*, *and g4stopping*) were utilized for all simulations. Range cuts of 20 mm were placed on secondary electrons and gammas, while a 0.05 mm range cut was placed on secondary protons. All simulations were carried out using the computation assistance of the University of Wisconsin- Madison Center of High Throughput Computing (CHTC) cluster.

**Figure 2.**
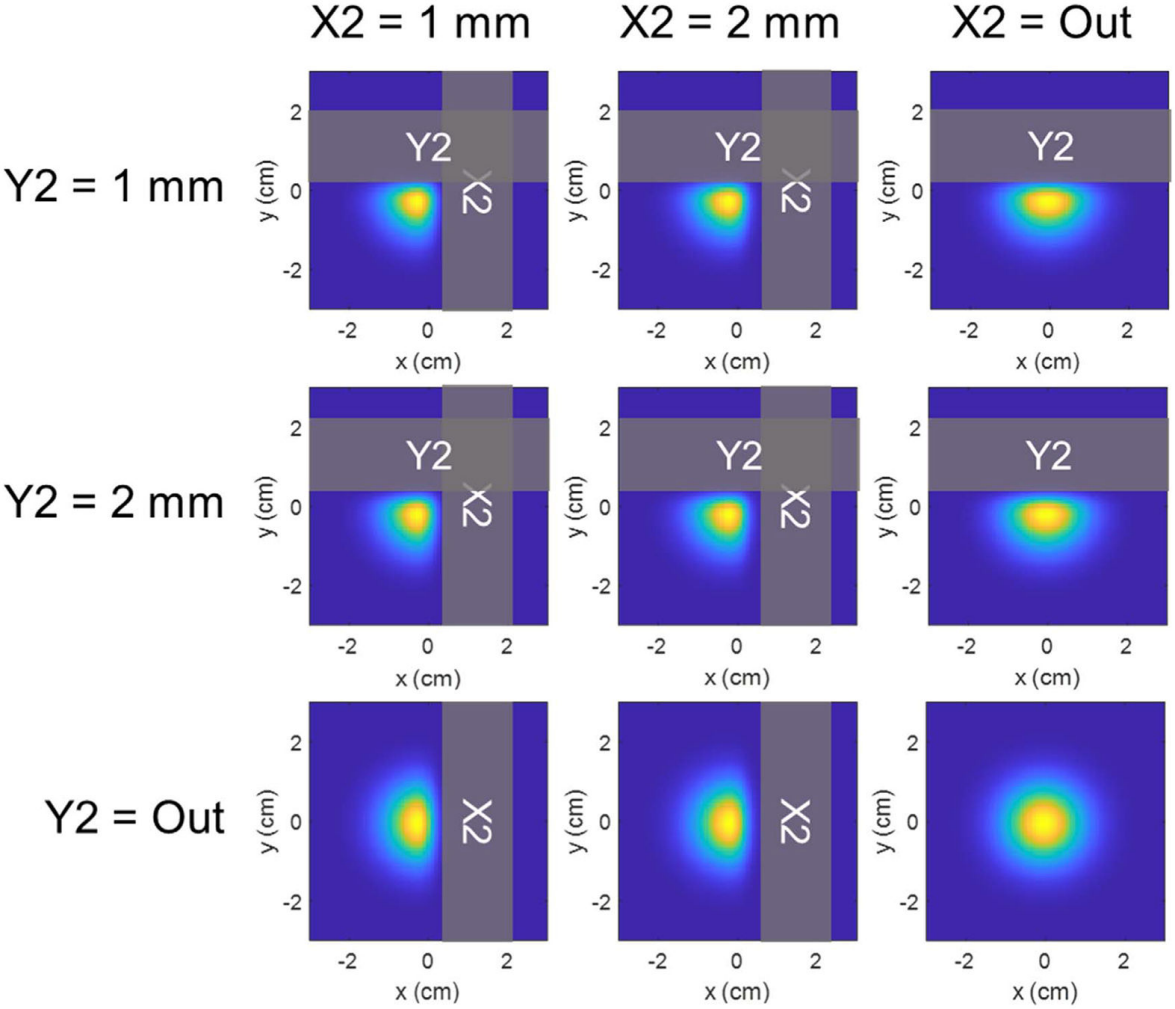
Simulated 78.3 MeV beamlet dose profiles for the trimmer configurations listed in table 2. Profiles are relative and displayed at the depth of the Bragg peak at 5 cm in water. DCS trimmers are overlaid in gray.

Following simulation of the X2- and Y2-collimated beamlets, two-dimensional (2D) dose distributions at the Bragg depth were analytically modified to account for collimation by all possible trimmer combinations (X1, X2, Y1, Y2), resulting in a total of 32 unique trimmed beamlets and one uncollimated beamlet for each energy. A set of the X2- and Y2-collimated beamlets at an energy of 78.3 MeV are illustrated in figure 2. These 2D beamlet libraries were then utilized to create and optimize uncollimated and collimated treatment plans for the targets in figure 1.

#### 2.1.2. Beamlet selection process

Uncollimated and collimated single energy treatment plans were created using a two-step process: beamlet selection and weight optimization. First, candidate beamlets were selected for a given target geometry based on a pre-defined dosimetric criteria, $DR_{thresh}$. Following the selection of candidate beamlets, a linear least squares weight optimizer was used to provide a uniform dose of 5 Gy to the target. To evaluate the dosimetric benefits of the DCS and enable equivalent comparisons between uncollimated and collimated treatments, the following 2D dose-area based criteria were maintained throughout the planning process:

1. Doses to 98% and 2% (e.g., D_98%_ and D_2%_) of the target areas must be within 3% of the prescription dose (4.85 and 5.15 Gy) for all plans.
2. Uncollimated and collimated D_98%_ and D_2%_ values must be within 1% of each other.

The beamlet selection algorithm proposed by Hyer *et al* was utilized to select beamlets for both uncollimated and collimated treatment fields, where beamlets are analytically shifted to model off-axis spots according to a pre-determined fixed spot map [7, 8]. For this work, all treatment plans utilized an in-plane spot spacing of 2.5 mm, with the exception the small kidney bean which utilized a 1 mm spot spacing because of the smaller size of the target. For each spot position, the dose ratio (DR) for each beamlet was calculated with respect to a pre-defined simplified conformity function, $DR_{thresh}$:

(1) $$DR=100\%\cdot \frac{D_{in}}{D_{out}}\geq DR_{thresh},$$

where $D_{in}$ is the total integrated beamlet dose inside the target and $D_{out}$ is the total integrated beamlet dose outside of the target. The $DR_{thresh}$ parameter largely influences the number of beamlets placed outside of the target for subsequent weight optimization. Following the beamlet selection algorithm, a weight optimization is carried out using a linear least squares optimizer developed by Flynn *et al* (2007) [32]. This optimizer considers point- and volume-based (or for this work, area-based) dosimetric penalties to define an optimization objective function. In general, the parameters used to define these objective functions were tuned to yield identical target coverage between the uncollimated and collimated cases.

**Figure 3.**
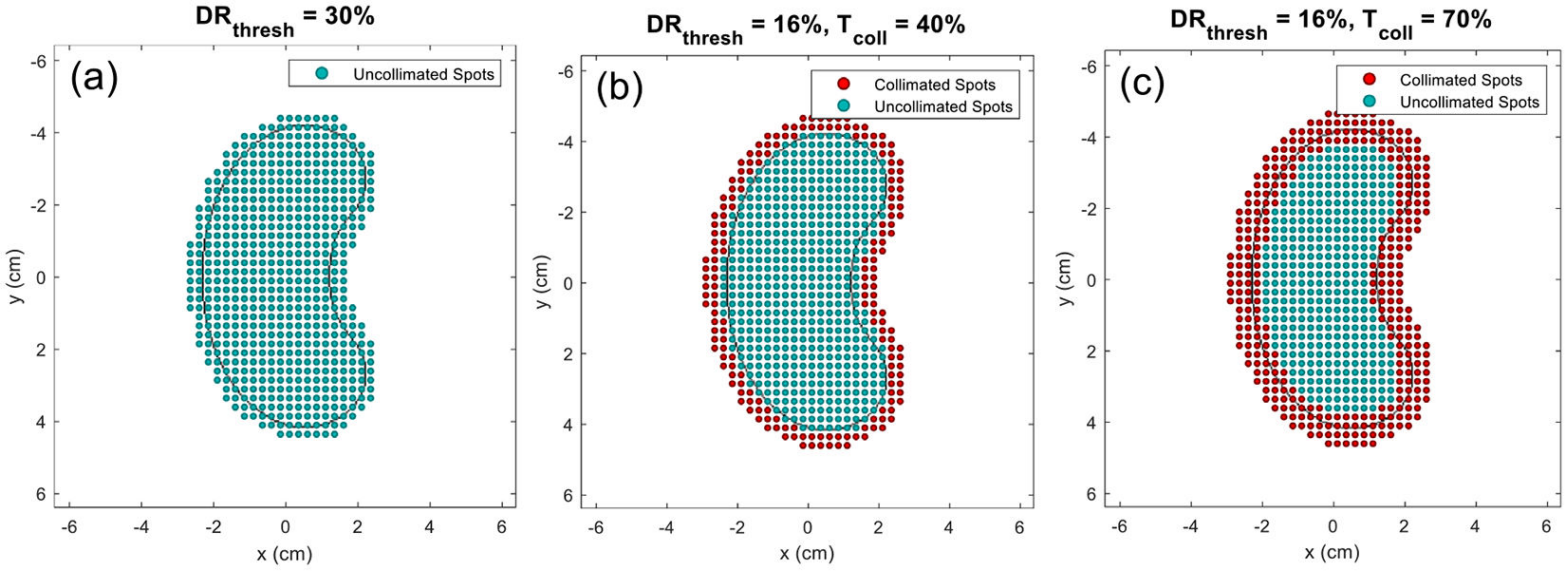
Spot maps for (a) uncollimated and (b and c) collimated large kidney bean treatments placed at a depth of 15 cm for collimation thresholds ($T_{coll}$) of (b) 40% (b) and (c) 70%. The uncollimated plan utilized a $DR_{thresh}$ value of 30% and both collimated plans utilized a $DR_{thresh}$ value of 16%.

For the creation of collimated fields, the number of collimated beamlets utilized in the plan, or level of collimation, must be decided. The level of collimation is dictated by a pre-defined threshold denoted as $T_{colb}$, which influences the number of collimated spots selected in each irradiation pattern. The influence of the $DR_{thresh}$ parameter is illustrated in figure 3 for uncollimated and DCS-collimated plans that utilized two differing levels of collimation ($T_{coll}=40\%$ and 70%) a depth of 15 cm. The influence of $T_{coll}$ is illustrated in the difference between figures 3(b) and (c). The mathematical definition of this parameter and its influence on DCS-collimated dose distributions is further discussed in the appendix. In general, however, increasing values of $T_{coll}$ increases the number of trimmed beamlets in the irradiation patterns. The influence of the level of collimation on deliverability was assessed experimentally at two levels for each treatment depth evaluated.

### 2.2. Mechanical integration

To enable the delivery of DCS-collimated treatment fields, modifications were made to the pencil layer definition (PLD) file, which is responsible for describing the irradiation patterns. The PLD file is converted into machine files for execution by the IBA Beam Delivery Control Unit (BDCU). The PLD file consists of layer blocks and elements, where layer blocks define the irradiation pattern for a specific energy layer, and elements contain information about the spot map and meterset weights (MU) for each spot. To incorporate trimmer positions, the PLD file was modified by adding the trimmer positions to the end of each element line. These trimmer positions were then transferred to a customized BDCU equipped with two I/O boards responsible for communicating the trimmer positions to the DCS control system. The DCS control system, in turn, provides feedback to the BDCU, enabling it to determine when the trimmers are correctly positioned. Once the trimmers are in place, the BDCU continues its normal operation and delivers the spot according to the specified MU.

### 2.3. Experimental dosimetry

Experimental validation was conducted to verify the accuracy and deliverability of DCS-collimated treatment plans. The validation focused on large kidney bean plans targeting depths of 5, 10, and 15 cm without the range shifter (NRS), as well as 5 cm with the range shifter (RS). To ensure accurate measurements, planar 2D dose distributions were obtained in the plateau region proximal to the Bragg peak to avoid dosimetric uncertainties near the peak. The measurements were performed in water using the MatriXX-PT (IBA Dosimetry, Schwarzenbruck, Germany) [34, 35] ionization chamber array and EBT3 radiochromic film (Ashland Specialty Ingredients, Bridgewater, NJ, USA) within in the DigiPhant PT scanning water tank. For 10 and 15 cm depth plans, measurements were taken at depths of 6 and 11 cm, respectively. For the 5 cm depth plans, measurements were performed at a depth of 4 cm, the shallowest possible measurement plane within the water tank. Central axis dose measurements were also conducted at the reference depths of 6 and 11 cm using the IBA PPC05 reference class ionization chamber. However, it was not feasible to perform PPC05 measurements at the 4 cm depth due to geometrical constraints.

The primary objective of these measurements was to validate the automated deliverability of DCS-collimated treatment plans in comparison to the simulated dose distributions and absolute dosimetry modeling achieved through Monte Carlo treatment planning. The accuracy of the Monte Carlo code’s dose calibration, expressed as Gy/proton, was verified by comparing it to the measured dose in Gy/MU. This comparison allowed for the determination of a Monte Carlo conversion factor in protons/MU under the reference conditions specified by IAEA TRS-398 [36]. These energy-specific conversion factors were then used to relate optimized weight in terms of protons to MU within the PLD delivery file.

Following the completion of the beamlet weight optimization, all plan parameters, including energy, spot positions, trimmer positions (if applicable), and number of MUs, were written as PLD files for delivery using a modified version of the IBA BDCU. The MatriXX measurements employed a dual irradiation technique, involving two measurements with a 3.8 mm shift to generate a dose distribution with a native resolution half that of the chamber-to-chamber spacing. The EBT3 films were scanned using an Epson^®^ Expression 10000XL (Epson America, Inc., Long Beach, CA) flatbed scanner at 300 dpi, which corresponds to a spatial resolution of 0.085 mm. All dose distributions were interpolated to a spatial resolution of 0.5 mm, and an optimized rigid registration was used to align the measured and simulated dose distributions through translations and rotations. The average central axis doses within the lateral dimensions of a PPC05 chamber were derived from the Monte Carlo and MatriXX-PT dose distributions. To assess agreement, absolute gamma analysis was performed between Monte Carlo and MatriXX-PT dose distributions, while relative gamma analysis was conducted between Monte Carlo and film dose to avoid LET saturation effects [37, 38].

While these measurements were performed with clinical tools, such as the MatriXX-PT, the absence of a spread-out Bragg peak distribution makes absolute dose verification more complicated in regions of dose gradients as a function of depth. Despite the measurements being performed proximal to the Bragg peak, the shallowest measurements performed at a depth of 4 cm were still in a relatively high-dose gradient region (5 %/mm) near the pristine Bragg peak at a depth of 5 cm. Because of this, a distance to agreement (DTA) in the depth direction was considered to account for any uncertainties associated with depth positioning. Planar 2D gamma analyses were carried out using criteria of 3%/3 mm, 3%/2 mm, and 2%/2 mm to evaluate the expected agreement range, however, the 3%/3 mm criterion is widely adopted clinically for PBS proton therapy [35, 39-41]. A dose threshold of 10% was used in all gamma analyses to compute the pass rate, following the recommendations of AAPM Task Group 218 for photon-based intensity modulated radiation therapy quality assurance (QA) [42]. There are currently no official recommendations for gamma dose thresholds for patient specific QA in proton therapy.

## 3. Results

### 3.1. Treatment planning

The uncollimated treatment fields were verified to have D_98%_ and D_2%_ values within 1.8% and 0.8% of the prescription dose, respectively, across all target geometries. In the case of collimated fields, the D_98%_ and D_2%_ values were verified to be within 1.5% and 0.8% respectively. Additionally, the differences in D_98%_ and D_2%_ values between the uncollimated and collimated plans were within 0.4% and 0.1% respectively. To achieve this agreement, $DR_{thresh}$ values of approximately 30% and 16% were required for the uncollimated and collimated plans respectively. The lower $DR_{thresh}$ value for collimated plans indicates that a larger number of beamlets were placed outside the target geometry compared to the uncollimated case. This is a logical result, additional beamlets were necessary to achieve the desired target coverage due to the sharper penumbra of the collimated beamlets.

### 3.2. Simulated dosimetric characterization

**Figure 4.**
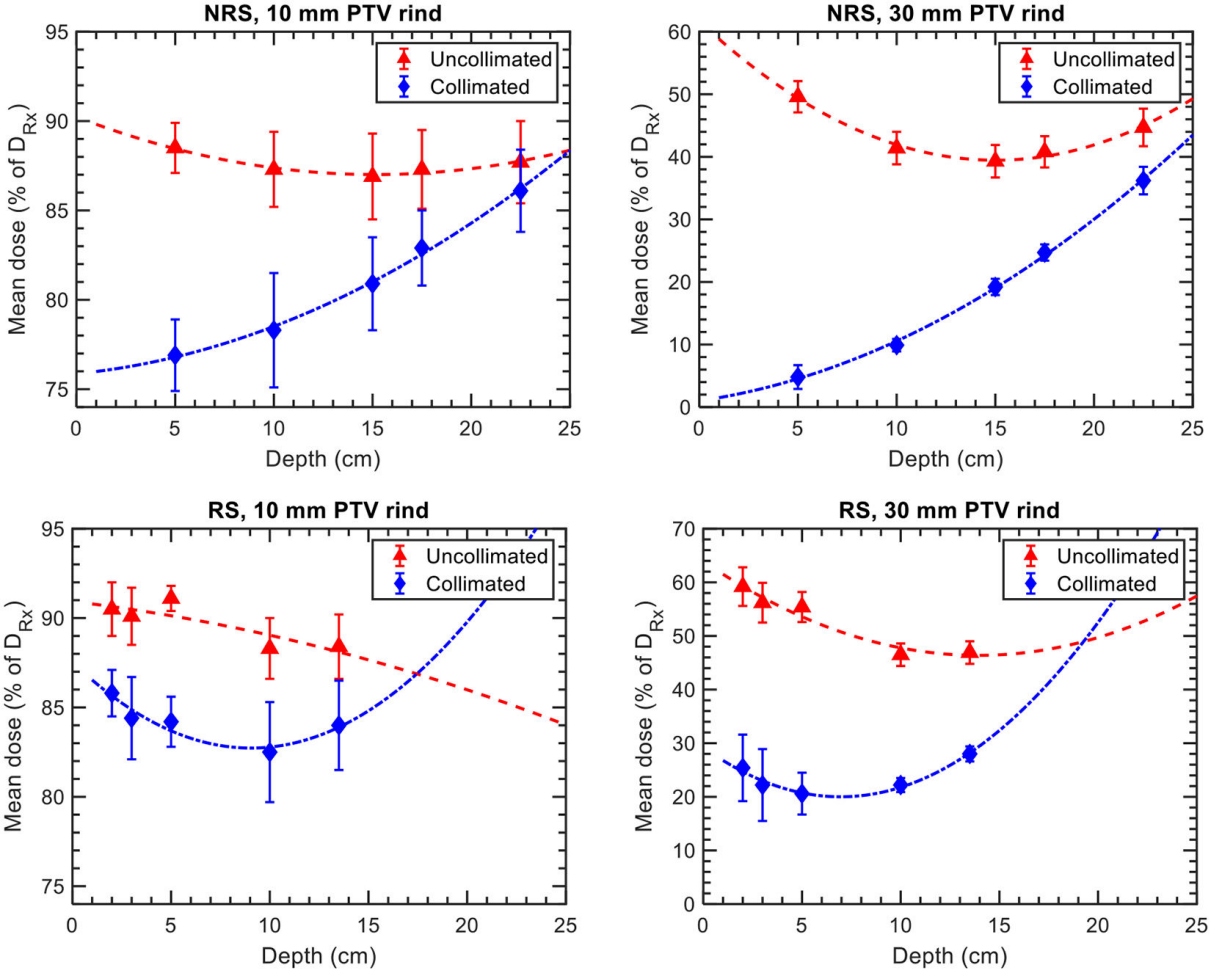
Plots of the mean dose, as a percentage of the prescription dose, as a function of depth for 10 (left column) and 30 mm (right column) rinds surrounding the targets for the NRS (top row) and RS (bottom row) plans evaluated *in silico*. Error bars correspond to the type A variation in mean doses across the circle and kidney bean targets evaluated.

**Figure 5.**
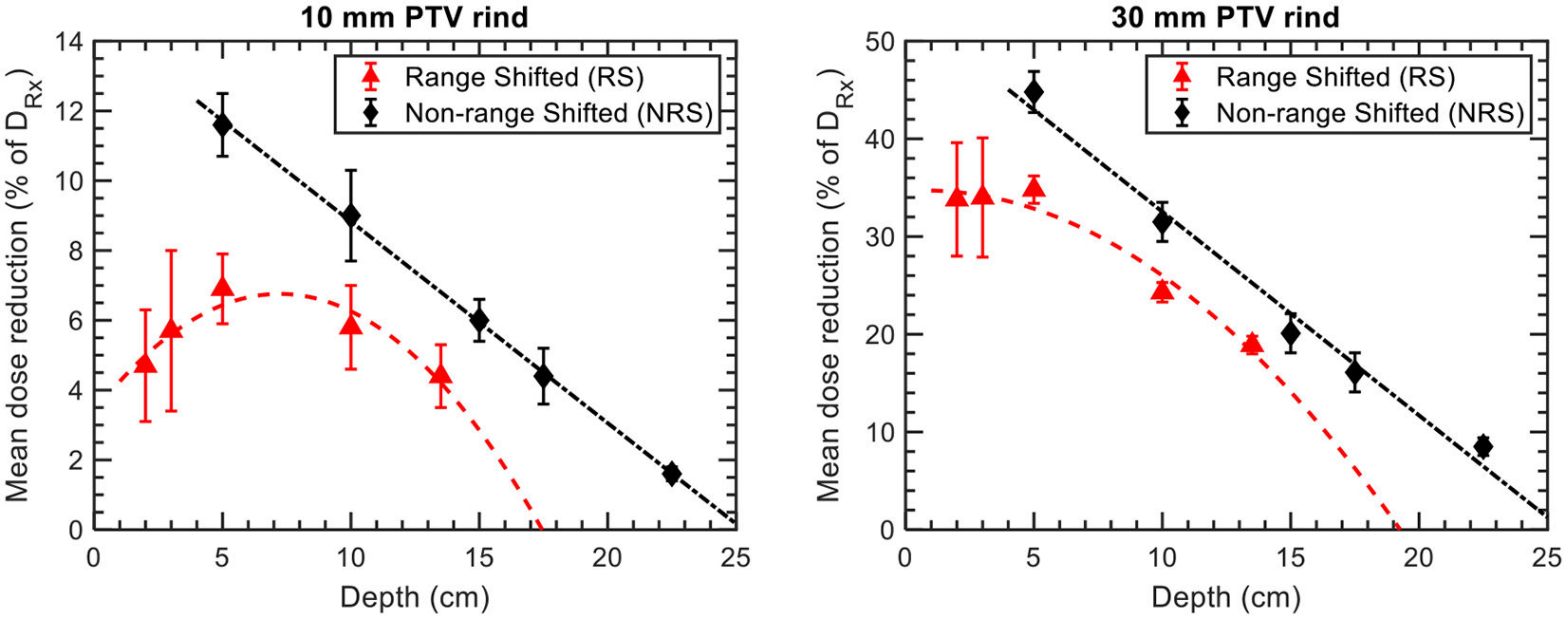
Plots of the reduction in mean dose, as a percentage of the prescription dose, as a function of depth for 10 (left) and 30 mm (right) rinds surrounding the targets for the NRS and RS plans evaluated *in silico*. Error bars correspond to the type A variation in the mean dose reductions across the circle and kidney bean targets evaluated.

To benchmark and evaluate the protection of normal tissues achieved by the DCS, we analyzed the mean dose (D_50%_) to 2D peripheral rind regions surrounding the target areas with widths of 10 mm and 30 mm. These doses were studied in relation to treatment depth and the presence of a range shifter. Figure 4 illustrates the mean doses to the examined regions for both uncollimated and collimated plans, considering cases that were either range shifted (RS) or non-range shifted (NRS). Additionally, figure 5 displays the dose reductions induced by collimation in the RS and NRS scenarios for the evaluated regions and targets. The results presented in figures 4 and 5 are averaged across the circle and kidney bean targets evaluated in figure 1, where the error bars correspond to the type A variation across all targets.

In general, the variations in target-specific dose reductions were more pronounced in the RS cases compared to the NRS cases. For both examined regions, the NRS plans exhibited a linearly decreasing dose reduction trend, while the RS cases demonstrated a non-linear behavior. In the NRS plans, the maximum simulated dose reductions for the 10- and 30-mm rinds were 11.6% ± 0.9% and 44.8% ± 2.1%, respectively, at a depth of 5 cm. These reductions linearly decreased at rates of 0.58% and 2.08% per cm in water, respectively. On the other hand, the RS plans showed lower simulated dose reductions compared to the NRS plans, with reductions of up to 6.9% ± 1.0% and 34.8% ± 1.4% at a depth of 5 cm for the 10- and 30-mm rinds, respectively. The theoretical depths at which the polynomial fits of the dose reduction data reach zero indicate the point at which the benefits of collimation are no longer present. For the 30 mm rind, these benefits are lost at depths of 17.5 cm and 22.5 cm in water for the RS and NRS cases, respectively. The presented results were obtained using $T_{coll}$ values of 65%–70%, depending on the treatment depth. The impact of this parameter on the sparing of normal tissues is discussed further in section 4.A. and the appendix.

#### 3.2.1. Trimmer-to-surface dependence

**Figure 6.**
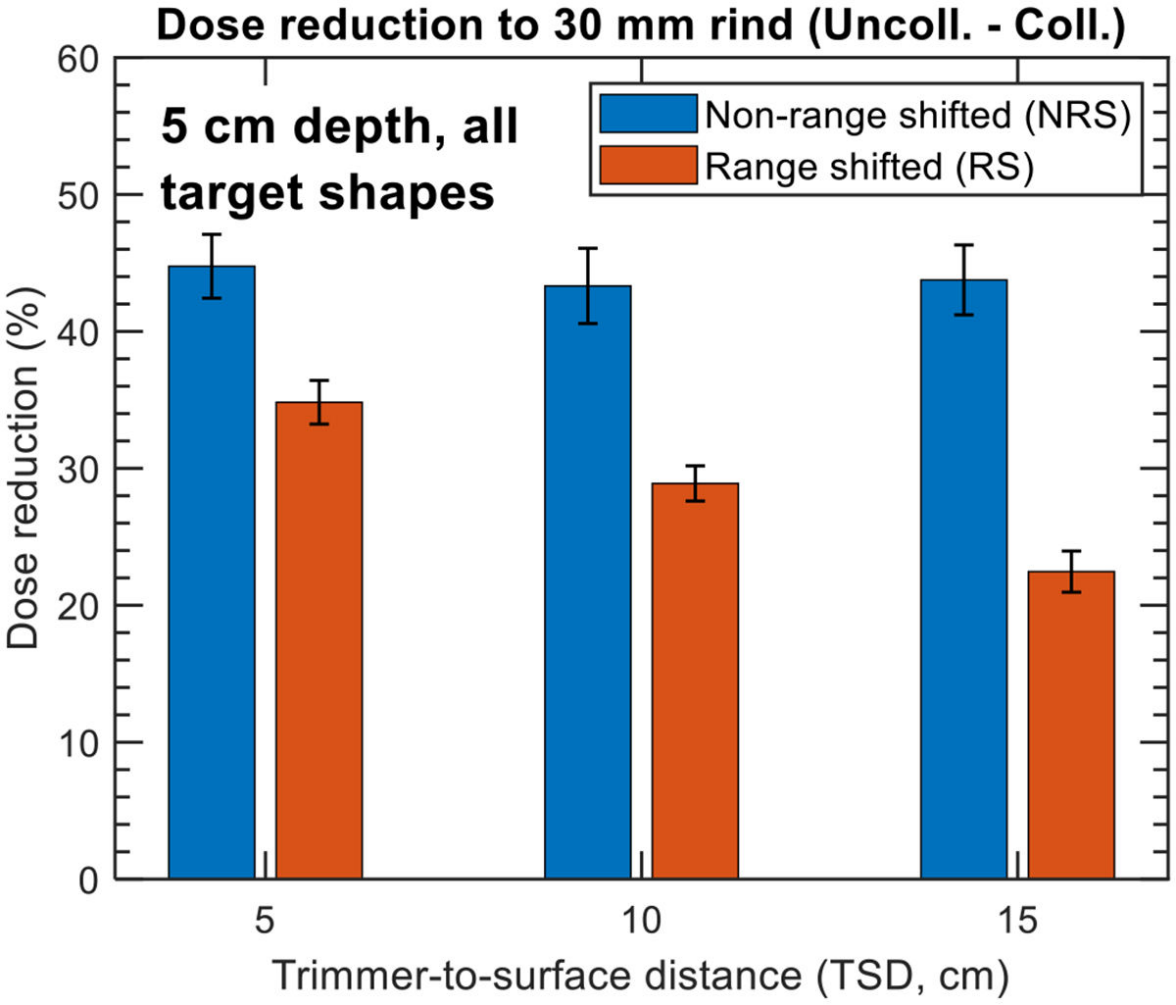
Bar plot of simulated collimation-induced reduction in mean dose to 30 mm normal tissue rind across the circle and kidney bean targets and TSDs evaluated for NRS and RS treatments at a depth of 5 cm in water. The error bars correspond to the standard deviation of the dose reduction averaged across all target shapes.

**Figure 7.**
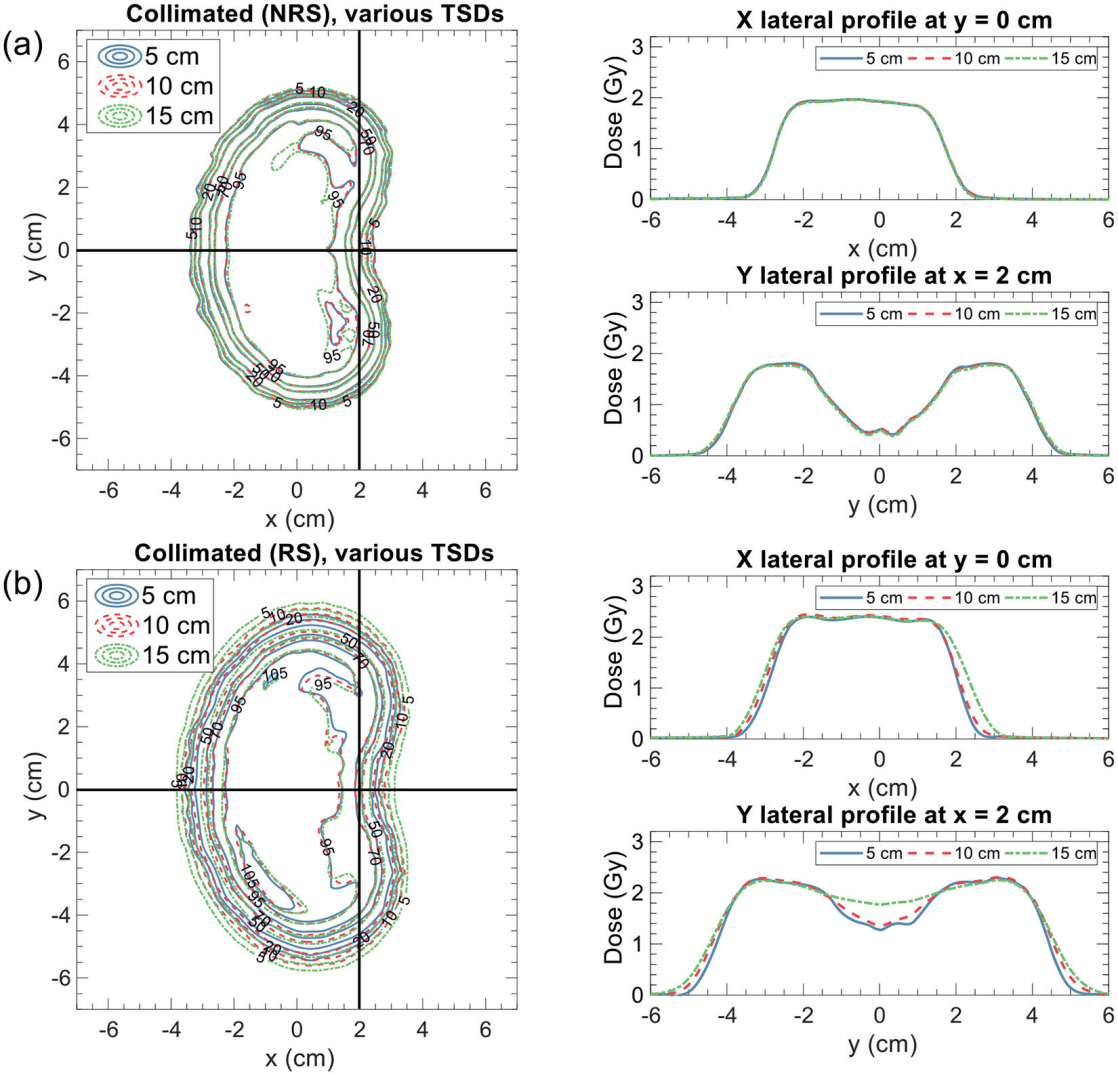
Comparison of MatriXX measurements for the 5 cm (a) NRS and (b) RS collimated kidney bean treatment plans for TSDs of 5, 10, and 15 cm.

Figure 6 displays the simulated reductions in mean dose to the 30 mm normal tissue rind, averaged across the RS and NRS cases at a depth of 5 cm for the range of trimmer-to-surface distance (TSD) values evaluated (5, 10, and 15 cm). For the RS cases, a strong dependence on the TSD is observed, with a 12.3% improvement in dose reduction observed when reducing the TSD from 15 to 5 cm, resulting in dose reductions of 22.5% and 34.8% respectively for the RS cases. The impact of the TSD is further demonstrated in figure 7, which presents MatriXX measurements for the collimated 5 cm NRS and RS kidney bean plans at TSDs of 5, 10, and 15 cm. Figure 7(b) specifically highlights the benefit of minimizing the TSD to maximize normal tissue sparing in the RS case, while figure 7(a) shows that the measured dose distributions for the NRS case are largely independent of the TSD, confirming the results presented in figure 6.

### 3.3. Dosimetric validation

**Figure 8.**
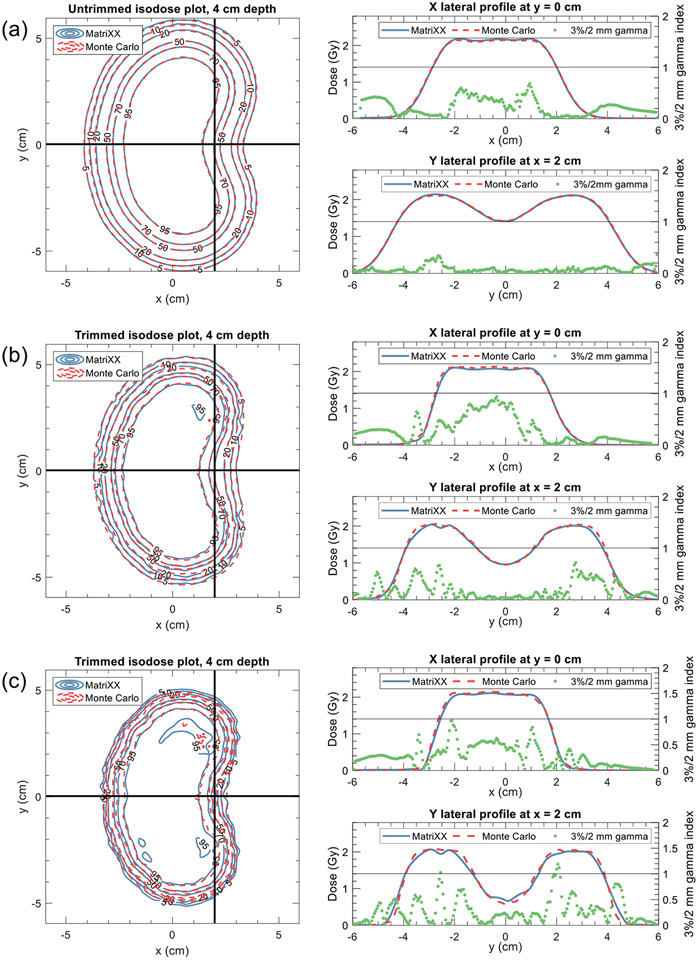
Comparison between Monte Carlo and MatriXX measurements at 4 cm depth for the large kidney bean treated at a depth of 5 cm for the uncollimated (a) and collimated cases (b & (c) with $T_{coll}$ values of 40% (b) and 65% (c). For each pane, relative isodose contour plots are displayed with dose profiles in the X- and Y-directions and corresponding 3%/2 mm gamma functions between Monte Carlo and MatriXX-measured dose points.

Table 3 presents the central axis doses obtained from Monte Carlo, MatriXX, and PPC05 measurements. In general, the PPC05 doses were within 2.2% of the Monte Carlo results for both trimmed ($T_{coll}=40\%$) and untrimmed plans on average. MatriXX doses were verified to within 1% of Monte Carlo on average, with average deviations of 1.2% for trimmed plans and 0.9% for untrimmed cases. The gamma analysis results for trimmed and untrimmed cases are provided in table 4. For the MatriXX-PT, gamma pass rates were computed for the trimmed cases that utilized $T_{coll}$ values of 40% and 70% or 65% (5 cm depth only). Overall, there was excellent agreement between Monte Carlo and film/MatriXX measurements. Using the 3%/2 mm criterion, the average 2D gamma pass rates were 99.7%, 99.1%, and 94.9% for untrimmed, lightly trimmed ($T_{coll}\;=\;40\%$), and heavily trimmed ($T_{coll}=65\%-70\%$) cases, respectively. Figure 8 illustrates the MatriXX and Monte Carlo results for untrimmed and trimmed cases at 5 cm depth (NRS). The depth-to-agreement (DTA) was maintained within 3 mm for all measurement configurations.

## 4. Discussion

### 4.1. Dosimetric characterization

For the simulated nominal treatment plans, average dose reductions of up to 45% and 35% were observed to the 30 mm rind were observed for the NRS and RS cases, respectively, for all targets evaluated. These results were obtained from plans that utilized $T_{coll}$ values of 65%–70%, which were later experimentally validated and compared to results from plans that utilized $T_{coll}$ values of 40%. The trade-off between dose reduction and deliverability for different $T_{coll}$ values is presented in table 5. Plans with a larger number of trimmer configurations showed reduced 3%/2mm pass rates compared to plans with fewer trimmer configurations. The choice of a specific $T_{coll}$ value may depend on the clinical context of the plan. For instance, at a depth of 5 cm, the substantial gain in dose reduction of 11.9% outweighed the minor decrease in gamma pass rate of 0.2%, favoring the plan with the larger $T_{coll}$ value. However, this trade-off may not be as desirable for the deeper case at a depth of 15 cm. While this investigation is not comprehensive enough to provide specific recommendations for $T_{coll}$ values, it offers experimental and computational results for various levels of trimming and demonstrates that this parameter has an impact on treatment planning and clinical use of the system.

It should be noted that the dosimetric results presented in this work were derived from single-energy treatment fields within a 2D space and are most representative of the distal energy layer within a target volume. For a multi-energy layer treatment plan, it is expected that the dose reductions in energy layers proximal to the most distal layer may slightly differ from the results presented in this work. The magnitude of this reduction would be dependent on the width of the SOBP and is the subject of future work within a clinical treatment planning system.

### 4.2. Experimental validation

Experimental validation was conducted for the large kidney bean plans at depths of 5, 10, and 15 cm in water. These experiments served to validate the plan creation process and the successful integration of the DCS and BDCU. Excellent agreement was observed between Monte Carlo simulations and measurements using the MatriXX-PT ionization chamber and EBT3 film. It is important to note that the dosimetric agreement reported include distance-to-agreement (DTA) in the depth direction, necessitated by the monoenergetic nature of the treatment plans. For the plans at 5 cm depth, measurements were taken at the shallowest possible depth in the DigiPhant water tank of 4 cm, which is in depth-dose gradient of 5 %/mm. The NRS and RS measurements were conducted using separate experimental setups of the equipment occurring at different times, which introduced some reproducibility uncertainty. As a result, the 3 mm depth DTA reported for the RS cases in table 4 accounts for systematic setup error compared to the depth DTAs reported for the NRS cases, which were 1 mm at most. These measurement conditions differ from typical clinical patient-specific quality assurance measurements, which are ideally performed in a uniform dose region to minimize variations caused by small setup uncertainties in the presence of depth-dependent dose gradients.

## 5. Conclusions

We have validated methods for creating and delivering DCS-collimated treatment fields using a Monte Carlo-generated beamlet library. Through this, we have parameterized the expected reductions in normal tissue dose with the DCS equipped to the IBA DN system and provided insights into treatment planning considerations. Lastly, we have performed dose verification for the fully automated delivery of dynamically collimated proton therapy for clinically relevant delivery patterns.

## Figures and Tables

**Table 1.** Summary of treatment depths evaluated in addition to the range shifter presence (RS = range shifted, NRS = non-range shifted), the beam energy, and spot size at isocenter for each respective depth.

Treatment depth (cm)	RS or NRS	Beam energy (MeV)	$\sigma_{air}$ (mm)
2	RS	86.9	7.6
3	RS	94.8	7.0
5	RS^a^	109.3	6.2
	NRS^a^	78.3	6.6
10	RS	140.5	4.9
	NRS^a^	116.0	5.0
13.5	RS	159.7	4.3
15	NRS^a^	146.1	4.2
17.5	NRS	159.7	3.8
22.5	NRS	184.6	3.4

a Indicates depths for which measurements were performed.

**Table 2.** Table of unique trimmer offsets considered in beamlet library. ‘Out’ indicates the trimmer was placed out of field and did not interact with the beamlet, resulting in an uncollimated final configuration.

Configuration #	1	2	3	4	5	6	7	8	9
X offset (mm)	1	1	2	2	Out	Out	1	2	Out
Y offset (mm)	1	2	1	2	1	2	Out	Out	Out

**Table 3.** Summary and comparison of Monte Carlo-simulated doses to MatriXX and PPC05-measured central axis doses for the untrimmed and trimmed NRS and RS large kidney bean measurements. The quantities in parentheses are the percent difference relative to Monte Carlo.

	Depth (cm)	Central axis dose (Gy)		
		Monte Carlo	MatriXX	PPC05
Untrimmed	5 (RS)	2.36	2.38 (0.9%)	N/A
	5 (NRS)	2.14	2.16 (0.9%)	N/A
	10 (NRS)	1.60	1.61 (0.7%)	1.63 (1.9%)
	15 (NRS)	1.82	1.84 (1.1%)	1.86 (2.1%)
Trimmed ($T_{coll}=40\%$)	5 (RS)	2.34	2.37 (1.3%)	N/A
	5 (NRS)	2.03	2.06 (1.5%)	N/A
	10 (NRS)	1.60	1.58 (−1.1%)	1.64 (2.6%)
	15 (NRS)	1.83	1.81 (−1.0%)	1.87 (2.3%)

**Table 4.** Gamma pass rate comparison for comparisons between Monte Carlo/EBT3 (relative) and Monte Carlo/MatriXX (absolute). Measurements for trimmed plans with $T_{coll}$ values of 65%–70% were performed with the MatriXX only. The DTA in depth is also tabulated for each measurement plane.

	Depth (cm)	Depth DTA (mm)	Criteria of gamma evaluation (10% dose threshold)					
			3%/3 mm		3%/2 mm		2%/2 mm	
			EBT3^a^	MatriXX	EBT3^a^	MatriXX	EBT3^a^	MatriXX
Untrimmed	5 (RS)	3	N/A	100	N/A	100	N/A	99.4
	5 (NRS)	1	100	100	100	100	100	99.1
	10 (NRS)	1	100	99.5	100	99.0	100	98.4
	15 (NRS)	1	100	100	100	99.8	100	98.7
	Average		**100**	**99.9**	**100**	**99.7**	**100**	**98.9**
Trimmed ($T_{coll}=40\%$)	5 (RS)	3	N/A	100	N/A	99.4	N/A	94.4
	5 (NRS)	0	100	99.3	100	98.5	100	88.1
	10 (NRS)	0	100	99.9	100	99.7	100	98.9
	15 (NRS)	0	100	99.6	100	98.9	100	97.8
	Average		**100**	**99.7**	**100**	**99.1**	**100**	**94.8**
Trimmed ($T_{coll}=65\%-70\%$)	5 (RS)	3	N/A	96.5	N/A	93.8	N/A	89.9
	5 (NRS)	1	N/A	99.3	N/A	98.3	N/A	92.0
	10 (NRS)	0	N/A	98.9	N/A	95.3	N/A	93.0
	15 (NRS)	1	N/A	96.1	N/A	92.2	N/A	89.8
	Average		**N/A**	**97.7**	**N/A**	**94.9**	**N/A**	**91.2**

a Indicates only relative gamma analysis was performed.

**Table 5.** Comparison of dose reduction to 30 mm rind for two levels of trimming ($T_{coll}$) evaluated and the respective and 3%/2 mm gamma pass rates between Monte Carlo and MatriXX measurements at depths of 5, 10 and 15 cm. The 5 cm (RS), 10 cm (NRS), and 15 cm (NRS) conditions utilized $T_{coll}$ values of 40% and 70% while the 5 cm (NRS) condition utilized $T_{coll}$ values of 40% and 65%.

Depth (cm)	Lightly trimmed ($T_{coll}=40\%$)		Heavily trimmed ($T_{coll}=65\%-70\%$)	
	Dose reduction (%)	Gamma pass rate (%)	Dose reduction (%)	Gamma pass rate (%)
5 (RS)	28.5	99.4	33.4 (↑ 4.9%)	93.8 (↓ 5.6%)
5 (NRS)	31.6	98.5	43.5 (↑ 11.9%)	98.3 (↓ 0.2%)
10 (NRS)	22.8	99.1	30.7 (↑ 7.9%)	95.3 (↓ 3.8%)
15 (NRS)	15.1	98.3	18.3 (↑ 3.3%)	92.2 (↓ 6.1%)

**Figure A1.**
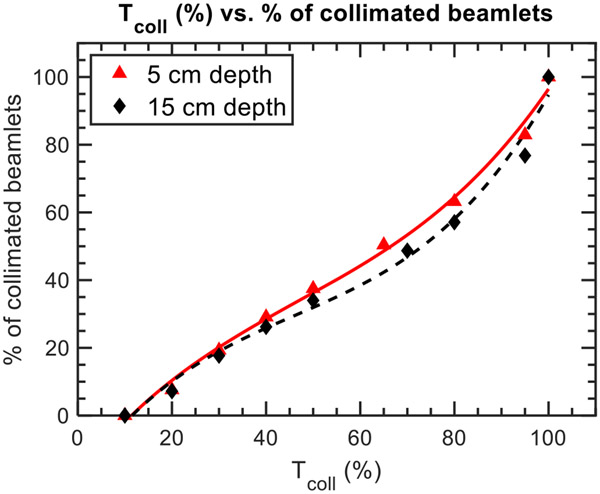
Plots of the relation between the $T_{coll}$ parameter and the number of collimated beamlets in a given irradiation pattern. The data presented are derived for treatment depths of 5 and 15 cm for the large kidney bean target.

**Figure A2.**
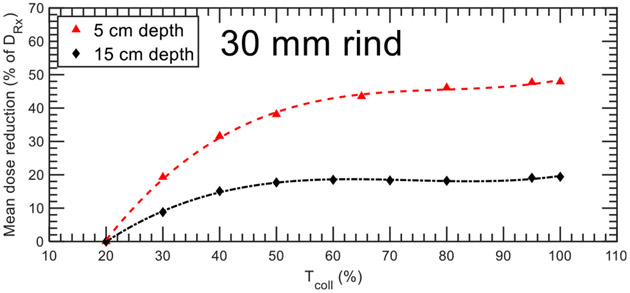
Plots of the reduction in mean dose to a 30 mm rind surrounding the large kidney bean target, as a percentage of the prescription dose, as a function of $T_{coll}$ for the NRS large kidney bean plans evaluated at depths of 5 and 15 cm *in silico*.

## Data Availability

All data that support the findings of this study are included within the article (and any supplementary files).

## Appendix

In the case of collimated planning with the DCS, an additional step must be taken within the beamlet selection algorithm to select the optimal collimated beamlet kernels, or trimmer configurations, that will be passed to the weight optimizer. Since the DCS is primarily used to collimate beamlets near the periphery of the target, relying solely on the proposed method for selecting collimated beamlets may result in an inefficient delivery. This means that the algorithm might place a significant number of collimated beamlets completely inside the target, providing little to no dosimetric benefit at the expense of increasing treatment time. To address this issue and avoid placement of collimated kernels inside of the target, a parameter ($T_{coll}$) is introduced within the beamlet selection algorithm. The fractional amount of total dose inside the target for the uncollimated beamlet kernel, $DR_{in,uncoll}$, is evaluated against another dosimetric threshold, $T_{coll}$, to decide whether a beamlet should remain uncollimated or not under the conditions using the following criterion:

(A1)
$$DR_{in,uncoll}=100\%\cdot \frac{D_{in,uncoll}}{D_{total,uncoll}}>T_{coll},$$

where $D_{in,uncoll}$ represents the total dose integrated inside of the target, $D_{total,uncoll}$ is the total integrated dose of the uncollimated kernel, and $T_{coll}$ is the dosimetric threshold for selecting a collimated kernel. If the condition in equation (A1) is true, indicating that the majority of the beamlet dose is inside the target, the beamlet remains uncollimated, and the algorithm proceeds to the next spot. If the condition is false, meaning that more than the specified percentage of the beamlet dose is outside the target, the beamlet is considered a desirable candidate for collimation. Therefore, as $T_{coll}$ increases, the relative proportion of collimated beamlets in the irradiation pattern will increase. When equation (A1) is false, the beamlet selection algorithm initiates an iterative search among all available collimated kernels in the library to find the kernel that maximizes the beamlet’s dose ratio ($D_{in}$/$D_{out}$) following the methods of Smith *et al* [33]. Once the optimal collimated kernel is selected, the algorithm moves on to evaluate the next spot position.

In general, one would expect that increasing the number of collimation configurations would enhance the dosimetric advantages of the DCS, albeit potentially extending the treatment time. To assess this trade-off in terms of dosimetric advantage, trimmed NRS treatment plans were generated for the large kidney bean target, considering $T_{coll}$ values ranging from 20% to 100%. When $T_{coll}$ is set to 100%, equation (A1) will always be false, resulting in the selection of collimated beamlets for all spot positions. On the other hand, the lower limit of 20% yields the condition in equation (A1) to always be true for the evaluated beamlets and target geometries, leading to an uncollimated treatment plan. Throughout this process, the dose-area criteria outlined in section 2.A.2 were maintained.

Figure A1 illustrates the percentage of total beamlets that underwent collimation as a function of the collimated dose threshold ($T_{coll}$) for the collimated treatment plans. Measurements were performed for plans with $T_{coll}$ values of 40% and 70% at depths of 10 and 15 cm, or 65% at a depth of 5 cm. The results of these measurements can be found in table 4 and further evaluated in table 5.

Figure A2 depicts the relationship between the $T_{coll}$ planning parameter and the dose reduction in the 30 mm rind surrounding the large kidney bean target at depths of 5 and 15 cm. In general, an asymptotic behavior was observed at both depths, where normal tissue dose reductions plateaued at 45% and 18% at depths of 5 cm and 15 cm, respectively. For the 15 cm depth, $T_{coll}$ values greater than 50% provided little to no dosimetric advantage. At a depth of 5 cm, $T_{coll}$ values exceeding 65% provided little to no dosimetric advantage.
